# mCSM-AB: a web server for predicting antibody–antigen affinity changes upon mutation with graph-based signatures

**DOI:** 10.1093/nar/gkw458

**Published:** 2016-05-23

**Authors:** Douglas E.V. Pires, David B. Ascher

**Affiliations:** 1 Centro de Pesquisas René Rachou, Fundação Oswaldo Cruz, Belo Horizonte 30190-002, Brazil; 2 Department of Biochemistry, University of Cambridge, Cambridge CB2 1GA, UK; 3 Department of Biochemistry, University of Melbourne, Victoria 3010, Australia

## Abstract

Computational methods have traditionally struggled to predict the effect of mutations in antibody–antigen complexes on binding affinity. This has limited their usefulness during antibody engineering and development, and their ability to predict biologically relevant escape mutations. Here we present mCSM-AB, a user-friendly web server for accurately predicting antibody–antigen affinity changes upon mutation which relies on graph-based signatures. We show that mCSM-AB performs better than comparable methods that have been previously used for antibody engineering. mCSM-AB web server is available at http://structure.bioc.cam.ac.uk/mcsm_ab.

## INTRODUCTION

Antibodies (Abs) are a central component of our immune system, generally recognizing a given antigen through variable loops of β-strands known as the complementarity determining regions (CDR's). This allows Abs to bind to a wide range of targets with high specificity, including those typically viewed as undruggable, which has been widely exploited experimentally, diagnostically and therapeutically. Mutations within the CDR's are important for determining the Abs specificity and affinity, however, like any therapeutic treatment, they can exert a selective pressure leading to the development of escape mutations, which are mutations typically located in the antigen, leading to reduced Ab-binding affinity. Engineering Abs often requires optimization of not just selectivity and affinity for the given antigen, but also stability, solubility and immunogenicity of the Ab (1). Optimization of these properties is essential not only for therapeutic use (2,3), but to improve quality and reproducibility in experimental settings (4).

A number of computational methods have been used to design and optimize Abs (5–8), normally using an available crystal structure, however accurate prediction of the effect of a mutation on the free energy of protein binding is non-trivial. This was highlighted recently by Sirin *et al*. who compiled an experimental dataset to benchmark these available methods, showing that there is still significant room for improvement, with the best methods only able to identify a third of mutations that improved binding. We have previously shown that using graph-based signatures to represent the 3D wild-type physicochemical and geometrical environment of a residue we could accurately predict the effects of mutations on protein stability, protein–protein affinity, protein–nucleic acid affinity and most recently protein–small molecule affinity (9–12). These have provided valuable insights into the effects of mutations in a variety of biological scenarios (13–16).

An accurate, robust and scalable computational approach would have enormous implications for not only directing Ab development, but in understanding the evolution and treatment of escape mutations, including through optimized vaccine design. We have therefore benchmarked our existing general methodologies against computational approaches for Ab engineering, and trained a novel Ab-specific predictor using the mCSM graph-based signatures concept in order to account for the unique and highly flexible recognition of Abs: mCSM-AB.

## MATERIALS AND METHODS

### Datasets

To assess the applicability of mCSM signatures in predicting the impact of mutations on Ab–antigen affinity, a dataset derived from the AB-Bind Database was considered (17). AB-Bind Database is a collection of experimental thermodynamic parameters for wild-type and mutant Abs and antigens, including the change in Gibbs free energy of binding (ΔΔ*G*), linked to published crystal structures of the complexes. A total of 645 single-point mutations on 29 different Ab–antigen complexes were considered, five of which were homology models, kindly provided by the AB-Bind database authors. Supplementary Figure S1 of Supplementary Data shows the experimental ΔΔ*G* distributions for the mutations in this dataset, which is skewed towards mutations that destabilize Ab-binding affinity. This is a limitation that affects the development of machine learning methods. In order to avoid any bias caused by this, within the training and test sets we have included models of the mutations (obtained using Modeller (18)) in order to consider the hypothetical reverse mutation (mutant to wild-type). This approach was initially proposed by (19) in order to better balance experimental observations where there is natural bias in the distribution of experimental observations, avoiding the subsequent bias in the computational models.

### Low-redundancy datasets

In order to reduce the chance of overfitting while training the predictive models and enhance their generalization, a procedure for reducing redundancy between cross validation folds was employed. Training and test sets for each fold were divided in a way that all mutations in a given residue position would only be present in either training or test set. The resulting low-redundancy sets are available at http://structure.bioc.cam.ac.uk/mcsm_ab/data.

### Graph-based structural signatures

**Figure 1.**
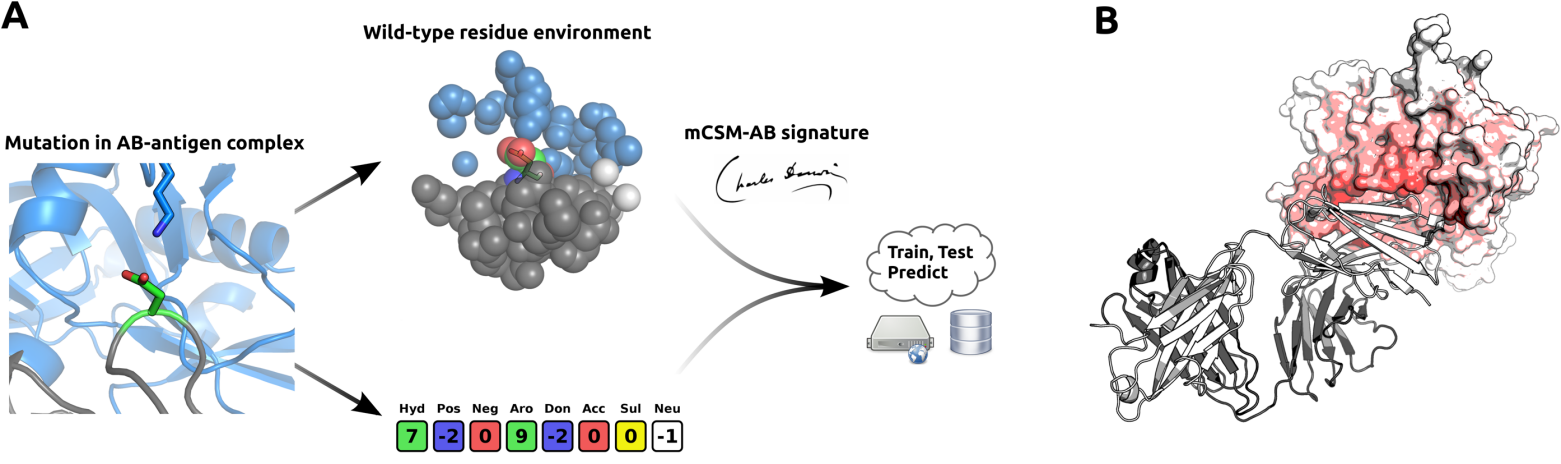
mCSM-AB workflow and application. (**A**) from a single-point mutation on a Ab-antigen complex, mCSM-AB extracts the wild-type residue environment from which a structural signature will be derived as well as a pharmacophore count difference between wild-type and mutant residue. These information, together with experimentally measured effects of mutations on Ab-antigen affinity from the literature are used as evidence to train and test a predictive model using machine learning. (**B**) shows a heatmap of predicted effects by mCSM-AB of all mutations on the complex formed by the VRC01 Ab and the HIV-1 gp120 (based on the PDB: 3NGB). Residues are coloured from white to red, with red being the residues with lower average prediction (most destabilizing ones).

Our approach uses a graph-based structural signature for Ab–antigen complexes that models both the geometry and physicochemical properties of the interactions and architecture of the wild-type Ab–antigen complex by representing atoms as nodes and interactions between them as edges. From this representation, distance patterns between atoms categorized by their properties are summarized in concise signatures as cumulative distributions and used as evidence for machine learning methods. Figure 1A depicts the mCSM-AB prediction workflow. Machine learning methods, evaluation procedures and performance metrics used are described in Supplementary Data.

## WEB SERVER

We have implemented mCSM-AB via a user-friendly web server freely available at http://structure.bioc.cam.ac.uk/mcsm_ab. The server front-end was built using Bootstrap framework version 2.0, while the back-end was built in Python via the Flask framework (version 0.10.1), running on a Linux server.

### Input

As shown in the job submission interface (Supplementary Figure S1), mCSM-AB allows users to upload Ab–antigen complexes (in PDB format) and inform mutations (a single mutation or a list), on either the Ab or antigen, for which the impact on antigen affinity can be predicted. The mutation information is given as the residue position, wild-type and mutant residue codes in one-letter format and chain identifier. The predictions are performed as a regression task (numerical prediction of the difference in Gibbs free energy—ΔΔ*G*). In order to aid users to submit jobs to mCSM-AB and interpret its predictions, a help page has been implemented and is accessible via the top navigation bar.

### Output

**Figure 2.**
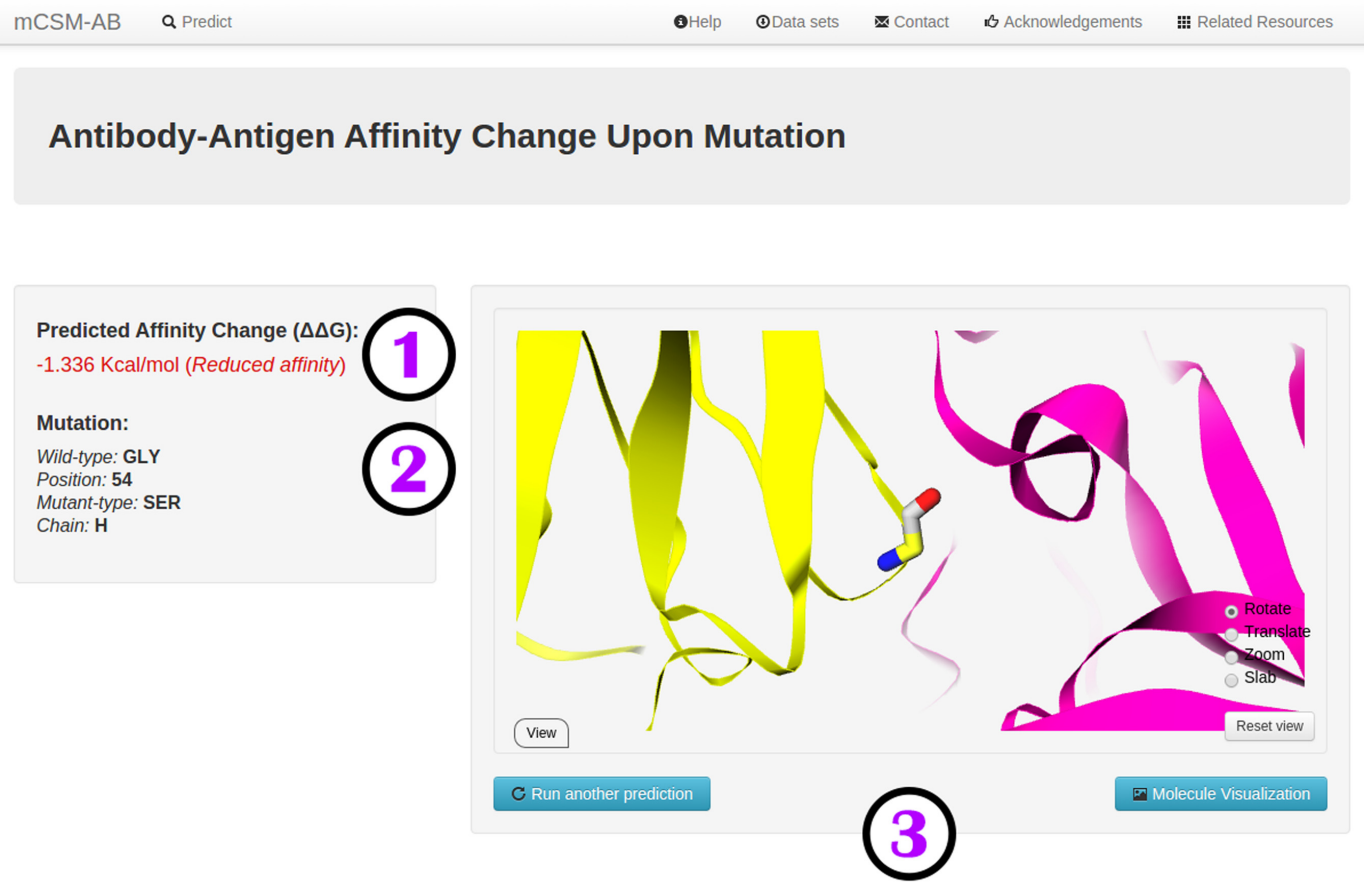
Web server result page. The figure depicts the result page for the single mutation prediction mode for mCSM-AB, which shows the numerical prediction (1), mutation information (2) and an interactive GLMol session of the residue structural location (3).

Figure 2 shows the result page for predicting the effect of a single mutation as a regression task. The predicted change in Gibbs Free Energy upon mutation (ΔΔ*G* in Kcal/mol) is given (i) as well as the identification of the provided mutation (ii) and a GLMol-based visualization of the mutated residue in the Ab–antigen structural environment (iii). A negative value (and red writing) corresponds to a mutation predicted as reducing affinity; while a positive sign (and blue writing) corresponds to a mutation predicted as increasing affinity. For classification tasks, the server will predict the mutation as either reducing or increasing affinity.

The result page for predicting effects of a list of mutations (Supplementary Figure S2) is given in a tabular format, containing the identification of the mutation, its residue relative solvent accessibility, predicted ΔΔ*G* or direction of change (increase/decrease affinity). An option to download the results as a tab-separated file is also available.

## VALIDATION

### Comparison with well established methods

As highlighted in Sirin *et al*. (17), the leading computational approaches for Ab engineering struggled to identify a majority of mutations leading to improved antigen affinity. We have previously published and characterized a machine learning method using graph-based signature to predict the effects of mutations on protein–protein binding affinities, mCSM-PPI (20). The benchmarking database used by Sirin *et al*. comprised of 29 Ab–antigen structures where the antigens were comprised of protein and peptide chains. We therefore evaluated the ability of mCSM-PPI to accurately predict the effects of mutations in these Ab–antigen complexes. mCSM-PPI performed as well as the leading methodologies analysed by Sirin *et al*., achieving a Pearson's correlation of *r* = 0.36. This highlights the ability of graph-based signatures to model the effects of mutations without the need to explicitly consider the effects of solvation, unlike methods relying on free energy perturbation, thermodynamic integration and empirical models where it needs to be considered for accuracy. mCSM-PPI is therefore significantly less computationally demanding, and can be run rapidly without the associated loss in accuracy.

Based on these promising results, we hypothesized that it might be more accurate and appropriate to build a graph-based methodology tailored specifically to consider the unique interaction interfaces presented by Abs (21), along with the more specialized cases where the antigen is a peptide (22–24), than a generalized PPI methodology where these types of interactions are underrepresented. We therefore built and trained a novel predictor: mCSM-AB.

**Figure 3.**
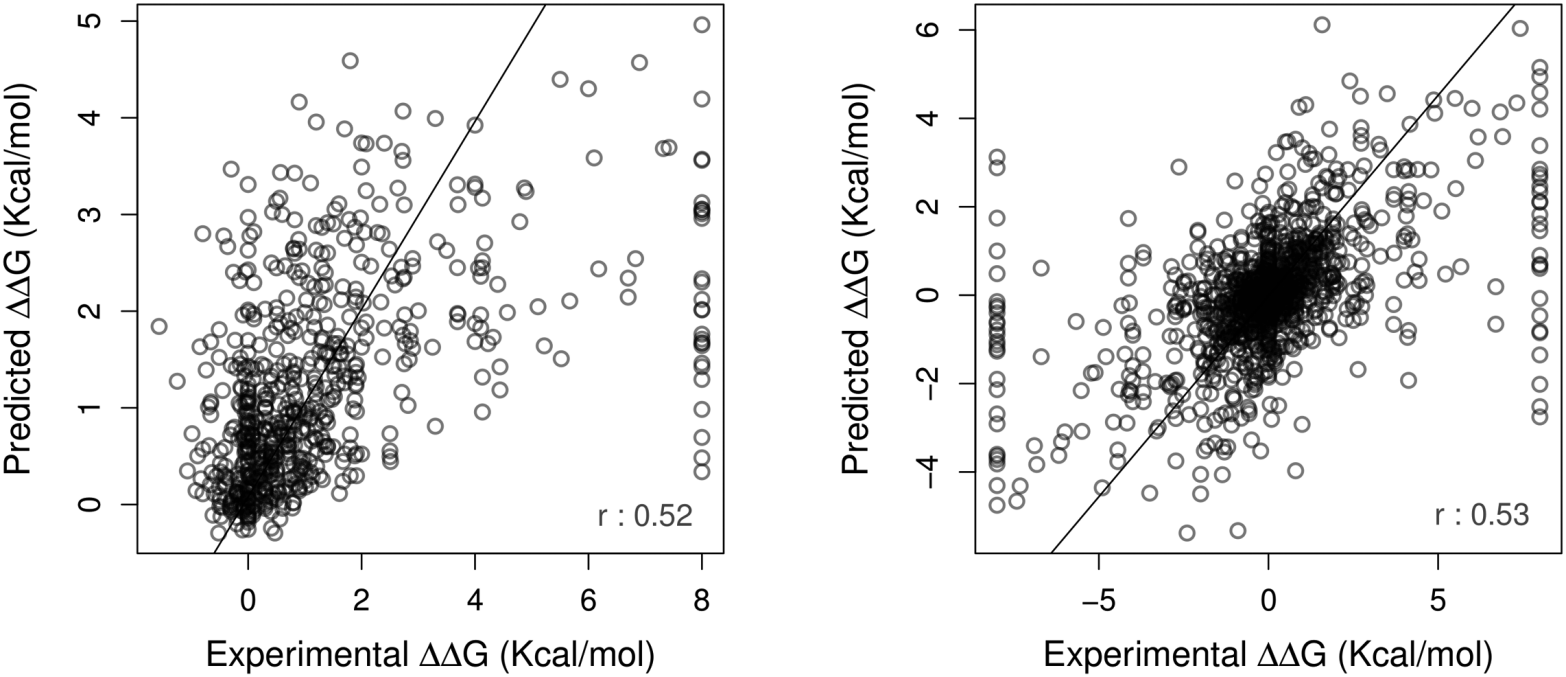
Regression plot between the experimental and predicted affinity change in Kcal/mol. mCSM-AB obtained a Pearson's correlation of 0.52 on the original dataset (left) and 0.53 on the dataset including hypothetical reverse mutations.

mCSM-AB performed significantly better than the methods evaluated by Sirin *et al*., achieving a Pearson's correlation of *r* = 0.53 on regression tasks on 10-fold cross validation, considering a low-redundancy dataset, with a standard deviation of 1.981. Figure 3 shows the regression plots between experimental and predicted affinity changes obtained for the original AB-Bind dataset (left-hand side) and for new method that also includes the models of the hypothetical reverse mutations (right-hand side). Table 1 presents a comparison of the performances between the considered methods. It is interesting to notice that the mCSM-AB was superior to most the compared methods, showcasing prediction capabilities of the proposed mCSM-AB platform.

**Table 1.** Performance comparison of available methods and mCSM-AB in classifying the direction of the change in Ab-antigen affinity caused by a mutation

Method	Pearson's coefficient
bASA	0.22
dDFIRE	0.19
DFIRE	0.31
STATIUM	0.32
Rosetta	0.16
FoldX	0.34
Discovery Studio	0.45
mCSM-PPI	0.35
**mCSM-AB**	**0.53/0.56^a^**

^a^Performance removing non-binders, variants determined not to bind within the sensitivity of the assay, for which ΔΔ*G* was set to −8 Kcal/mol (17).

The performance of the available methods are from Sirin *et al*. (17).

The mCSM-AB predictive model built using the hypothetical reverse mutations was more robust and accurate than the model built using just the experimental observations. While the Pearson's correlations were not statistically different from the model trained with the original dataset, its ability to differentiate mutations that enhance or reduce binding affinity was significantly improved.

During cross validation, mCSM-AB achieved correlations of 0.32 and 0.30 for mutations increasing and decreasing affinity, respectively. It is important to point out, that even though the correlations were lower than the complete dataset, mCSM-AB still correctly assigns the direction of the change for 77 and 76% of the increasing and decreasing affinity mutations, respectively.

### Blind test validation on homology models

In the original dataset proposed by (17), there were 5 Ab–antigen homology model complexes, containing 87 mutations in total. Of these mutations, ∼63% (55 mutations) lead to a decrease in experimental binding affinity, whilst 37% (32 mutations) had been shown experimentally to increase binding affinities. The hypothetical reverse mutations were also included, generating a balanced dataset. These were therefore removed from the mCSM-AB training set, which therefore was trained on experimental structures only, and used to evaluate the final models.

The model achieved a Pearson's correlation of 0.45 with the experimentally measured affinities of the homology modelled complexes. mCSM-AB presented a slightly lower performance than in cross validation, but with a lower standard deviation (1.30), which provides confidence in the applicability of this approach beyond experimental structures to those that are computationally modelled. Supplementary Table S1 shows the performance of mCSM-AB per homology model.

### Predicting HIV-1 escape mutations

We also wanted to evaluate the applicability of these models outside of experimentally measured affinities to biological systems, in particular the development of escape mutations that hinder the usefulness of therapeutic Abs. For this evaluation we used the anti-HIV therapeutic Ab VRC01, which recognizes the HIV-1 envelope glycoprotein gp120 complex. The effect of 78 distinct mutations upon the effectiveness of VRC01 had been studied in (25). Of these mutations, 33 increased and 45 decreased HIV-1 sensitivity to this neutralization Ab. Using the experimental crystal structure of the VRC01–p120 complex (PDB ID: 3NGB), we tested whether mCSM-AB could correctly classify those mutations which resulted in reduced effectiveness of VRC01. mCSM-AB predictions correlated strongly to the biological measurements, with a Pearson's correlation of 0.51 (and consistent with performance on the AB-Bind database). This highlighted the potential predictive power of this approach for exploring the consequences of biologically and clinically relevant mutations.

We therefore expanded our analysis by performing computational saturation mutagenesis of the entire gp120 structure and mapping the average predicted changes in binding affinity onto the structure (Figure 1B). This could be of significant help in identifying regions more likely to lead to develop escape mutations against Ab therapy, and help guide design and development strategies.

mCSM-AB has also been further validated on a dataset of 114 mutations on 4 Ab/antigen complexes (228, including hypothetical reverse mutations) from (26), achieving a correlation of 0.67, as described in Supplementary Data.

## CONCLUSIONS

We present here a new approach, mCSM-AB, which relies upon graph-based signatures to predict the impact of missense mutations upon the binding affinity of an Ab for a given antigen. The results achieved by mCSM-AB support the idea that the molecular shape and physicochemical complementarity, driving forces guiding molecular recognition on Ab–antigen complexes can be modelled and its determinant features mined with structural signatures. This allowed us to tailor a new method to accurately and robustly predict the effects of mutations. We believe that mCSM-AB will be a useful tool in the design and development of therapeutic and diagnostic Abs, and could provide useful insight into the development of escape mutations. A web server implementing mCSM-AB functionality is freely available at http://structure.bioc.cam.ac.uk/mcsm_ab.

## Supplementary Material

SUPPLEMENTARY DATA
