# Supplementary material for: mCSM-AB: a web server for predicting antibody–antigen affinity changes upon mutation with graph-based signatures
Source: Nucleic Acids Res. 2016 May 23;44(Web Server issue):W469–73. doi: 10.1093/nar/gkw458 (PMC4987957; doi:10.1093/nar/gkw458)
Supplement: SUPPLEMENTARY DATA [file supp_44_W1_W469__index.html]

mCSM-AB: a web server for predicting antibody–antigen affinity changes upon mutation with graph-based signatures — mCSM-AB: a web server for predicting antibody–antigen affinity changes upon mutation with graph-based signatures — SUPPLEMENTARY DATA 

# mCSM-AB: a web server for predicting antibody–antigen affinity changes upon mutation with graph-based signatures

## SUPPLEMENTARY DATA

- SUPPLEMENTARY DATA
