# Supplementary material for: mCSM-AB: a web server for predicting antibody–antigen affinity changes upon mutation with graph-based signatures
Source: Nucleic Acids Res. 2016 May 23;44(Web Server issue):W469–73. doi: 10.1093/nar/gkw458 (PMC4987957; doi:10.1093/nar/gkw458)
Supplement: SUPPLEMENTARY DATA [file supp_gkw458_nar-00276-web-b-2016-File010.pdf]

mCSM-AB: a web server for predicting antibody-antigen affinity changes upon mutation with graph-based signatures

Douglas E. V. Pires*, David B. Ascher*

Supplementary Material

1 Figures

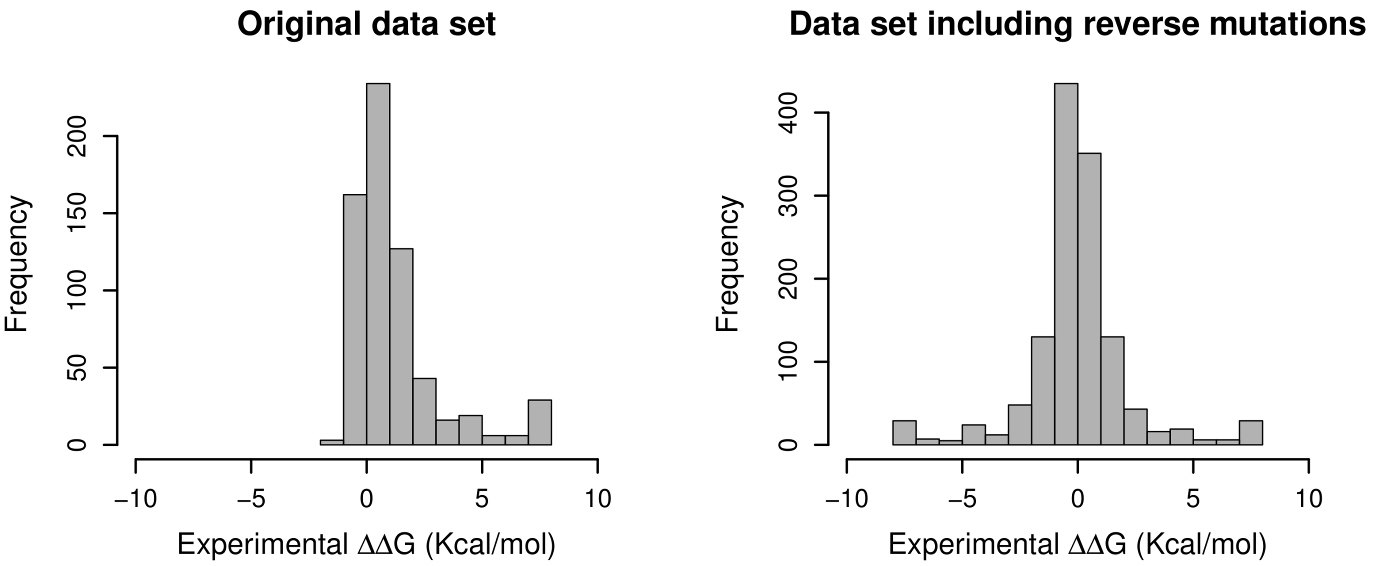

Figure 1: Distribution of experimentally measured effects of mutations on the mCSM-AB data set (given as the difference in Gibbs Free Energy - $\Delta\Delta G$). The histogram on the left shows the original distribution of the 645 mutations and the histogram on the right the histogram including the hypothetical reverse mutations.

2 Tables

*Email: douglas.pires@cpqrr.fiocruz.br; Correspondence may also be addressed to dascher@svi.edu.au

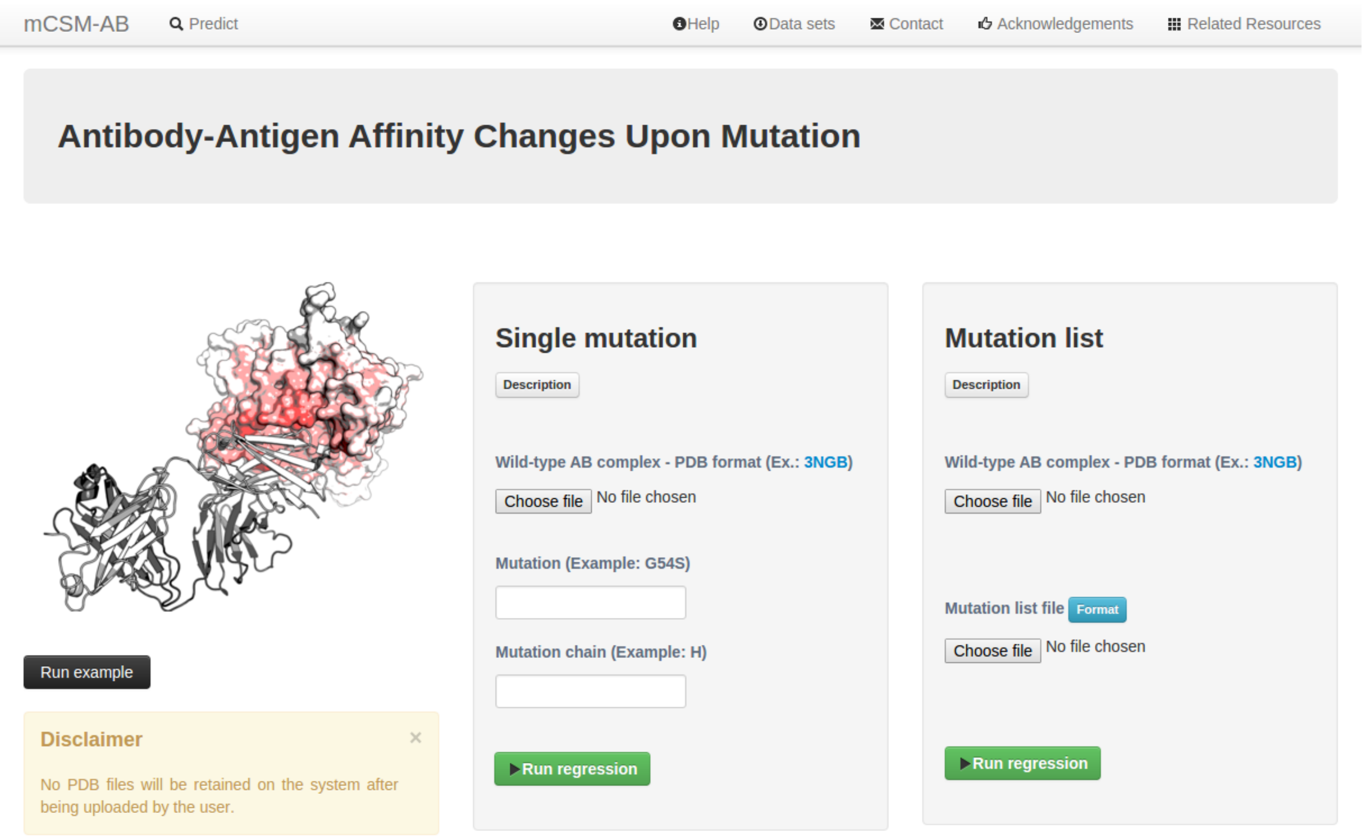

Figure 2: mCSM-AB job submission interface. Users have the option of predicting the effects of single mutations on an uploaded AB-antigen complex or alternatively a list of mutations. The predictions are performed as a regression task (numerical prediction of the difference in Gibbs Free Energy - $\Delta\Delta G$).

Table 1: Pearsons correlation coefficients obtained in blind test per homology model.

Homology model	Correlation	Correlation (Increased affinity)	Correlation (Reduced affinity)
HM_1KTZ	0.87	0.64	0.55
HM_1YY9	-0.04	-0.51	-0.46
HM_2NYY	0.48	0.60	0.24
HM_2NZ9	0.37	0.66	0.04
HM_3BN9	0.54	0.42	0.51
All models	0.45	0.44	0.22
All models except HM_1YY9	0.54	0.53	0.30

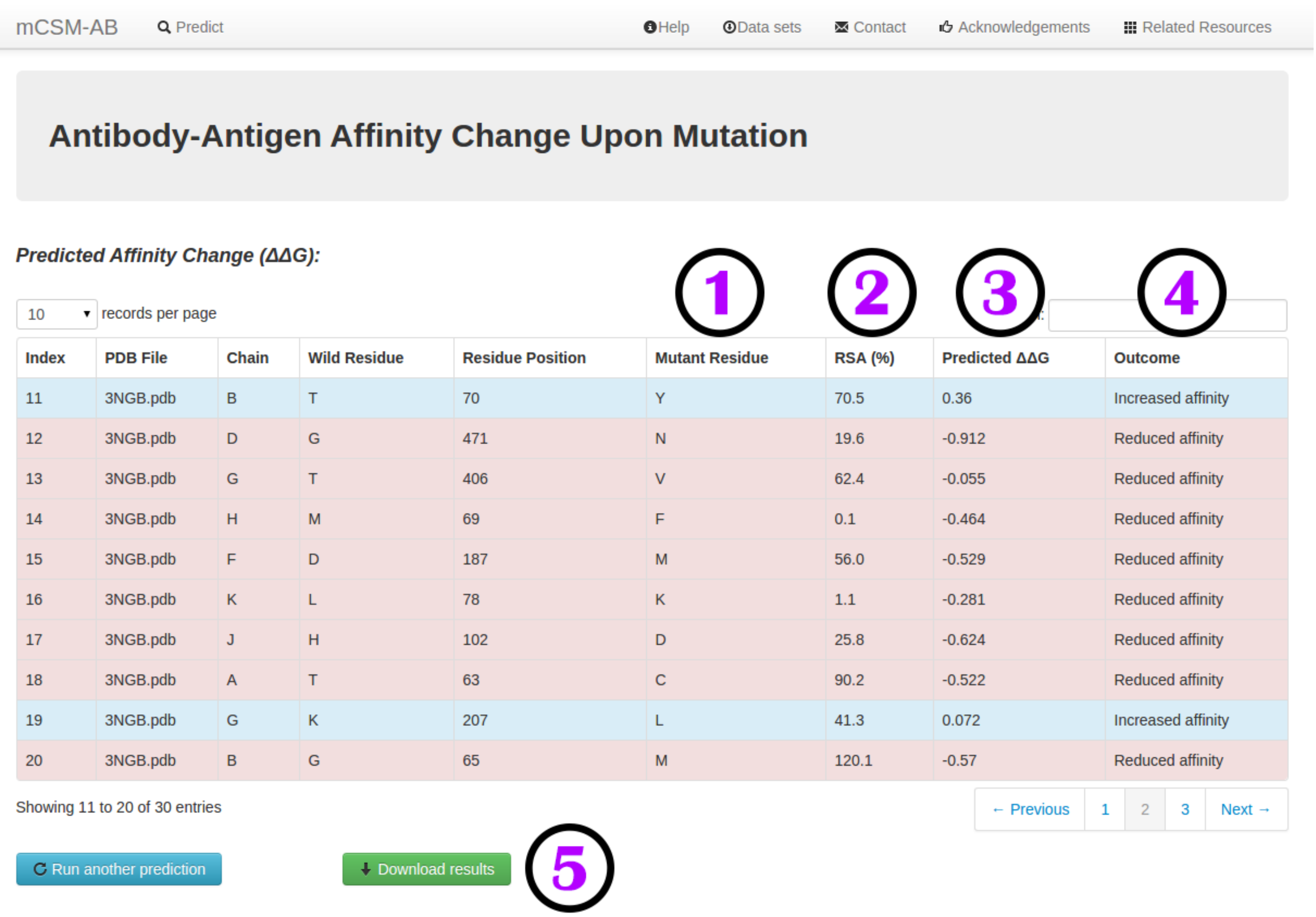

Index	PDB File	Chain	Wild Residue	Residue Position	Mutant Residue	RSA (%)	Predicted ΔΔG	Outcome
11	3NGB.pdb	B	T	70	Y	70.5	0.36	Increased affinity
12	3NGB.pdb	D	G	471	N	19.6	-0.912	Reduced affinity
13	3NGB.pdb	G	T	406	V	62.4	-0.055	Reduced affinity
14	3NGB.pdb	H	M	69	F	0.1	-0.464	Reduced affinity
15	3NGB.pdb	F	D	187	M	56.0	-0.529	Reduced affinity
16	3NGB.pdb	K	L	78	K	1.1	-0.281	Reduced affinity
17	3NGB.pdb	J	H	102	D	25.8	-0.624	Reduced affinity
18	3NGB.pdb	A	T	63	C	90.2	-0.522	Reduced affinity
19	3NGB.pdb	G	K	207	L	41.3	0.072	Increased affinity
20	3NGB.pdb	B	G	65	M	120.1	-0.57	Reduced affinity

Figure 3: mCSM-AB result page for predicting effects of a list of mutations as a regression task. The following information will be displayed: identification of the mutation (1), residue relative solvent accessibility (RSA) (2), Predicted $\Delta\Delta G$ (3) and direction of change (4). Users can also download the predictions as a tab-separated file (5).

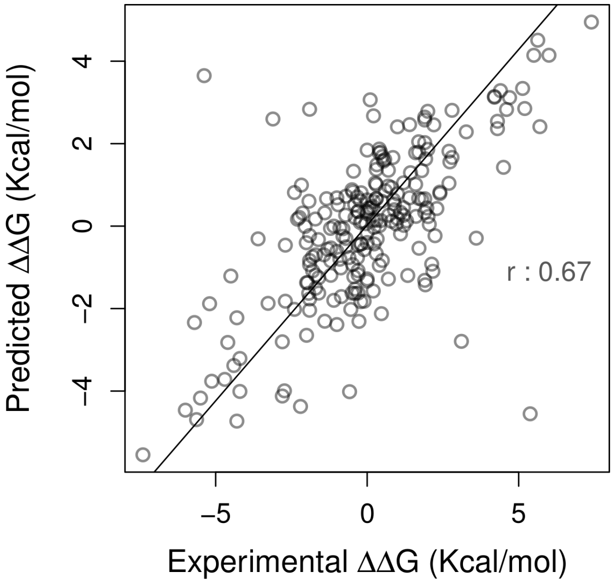

Figure 4: Regression plot between the experimental and predicted affinity change in Kcal/mol for a blind test derived from [1]. mCSM-AB obtained a Pearson's correlation of 0.67.

3 Case Study

We have further evaluate mCSM-AB oin a complementary blind test composed by 114 mutations from 4 different antibody/antigen, as well as their hypothetical reverse mutations obtained from [1].

The Figure S4 shows a regression plot between experimental and predicted affinity changes for the 228 mutations. mCSM-AB achieved a correlation of 0.67 on this blind test. The models and predictions are available as downloadable files on the Web server.

4 Supervised Learning

4.1 Regression

For regression tasks, the prediction goal for mCSM-AB was the change affinity of the Ab-antigen complex denoted by the variation in Gibbs Free Energy of binding ($\Delta\Delta G = \Delta G_{wt} - \Delta G_{mt}$) of a mutation, where ΔG_{wt} and ΔG_{mt} are the Gibbs Free Energy of binding for the wildtype and mutant complexes respectively. Different categories of algorithms implemented in the Weka toolkit [2] were evaluated. Those included K-Nearest Neighbours (KNN), Regression Trees and Gaussian Processes, with the latter presenting the best performance. Gaussian Process [3] is a flexible non-linear method for regression and data interpolation based on a kernel function, which also gives uncertainty estimation of the predictions.

4.2 Evaluation procedures and metrics

The predictive models' were evaluated under 10-fold cross validation and in blind tests. The following metrics were used to assess predictive performance and were chosen to directly compare mCSM-AB with the other available methods:

- **Pearsons correlation coefficient (r):** Used in regression tasks, quantifies que linear correlation/association between two variables, its direction and dispersion/strength. Its values range from -1 to 1, where perfectly correlated variables achieve an r of 1 (or -1, for a negative correlation) while a correlation of 0 would denote no linear relationship between variables.

References

[1] Alexander Benedix, Caroline M Becker, Bert L de Groot, Amedeo Caflisch, and Rainer A Böckmann. Predicting free energy changes using structural ensembles. *Nat. Methods*, 6(1):3–4, 2009.

[2] Mark Hall, Eibe Frank, Geoffrey Holmes, Bernhard Pfahringer, Peter Reutemann, and Ian H Witten. The WEKA data mining software: an update. *ACM SIGKDD explorations newsletter*, 11(1):10–18, 2009.

[3] Carl Edward Rasmussen and Christopher KI Williams. *Gaussian processes for machine learning*, volume 1. MIT Press, Cambridge, MA, USA, 2006.
